# Acute respiratory distress syndrome (ARDS): from mechanistic insights to therapeutic strategies

**DOI:** 10.1002/mco2.70074

**Published:** 2025-01-26

**Authors:** Rongli Xie, Dan Tan, Boke Liu, Guohui Xiao, Fangchen Gong, Qiyao Zhang, Lei Qi, Sisi Zheng, Yuanyang Yuan, Zhitao Yang, Ying Chen, Jian Fei, Dan Xu

**Affiliations:** ^1^ Department of General Surgery Ruijin Hospital Lu Wan Branch, Shanghai Jiaotong University School of Medicine Shanghai China; ^2^ Department of Urology Ruijin Hospital, Shanghai Jiaotong University School of Medicine Shanghai China; ^3^ Department of General Surgery, Pancreatic Disease Center Ruijin Hospital, Shanghai Jiaotong University School of Medicine Shanghai China; ^4^ Department of Emergency Ruijin Hospital, Shanghai Jiaotong University School of Medicine Shanghai China; ^5^ Department of Radiology Södersjukhuset (Southern Hospital) Stockholm Sweden; ^6^ Department of Molecular and Human Genetics Baylor College of Medicine Houston Texas USA; ^7^ Department of Radiology The First Affiliated Hospital of Zhejiang Chinese Medical University Hangzhou Zhejiang China; ^8^ Department of Immunology and Microbiology Shanghai Jiao Tong University School of Medicine Shanghai China

**Keywords:** ARDS, pneumonia, sepsis, severe acute pancreatitis, trauma

## Abstract

Acute respiratory distress syndrome (ARDS) is a clinical syndrome of acute hypoxic respiratory failure caused by diffuse lung inflammation and edema. ARDS can be precipitated by intrapulmonary factors or extrapulmonary factors, which can lead to severe hypoxemia. Patients suffering from ARDS have high mortality rates, including a 28‐day mortality rate of 34.8% and an overall in‐hospital mortality rate of 40.0%. The pathophysiology of ARDS is complex and involves the activation and dysregulation of multiple overlapping and interacting pathways of systemic inflammation and coagulation, including the respiratory system, circulatory system, and immune system. In general, the treatment of inflammatory injuries is a coordinated process that involves the downregulation of proinflammatory pathways and the upregulation of anti‐inflammatory pathways. Given the complexity of the underlying disease, treatment needs to be tailored to the problem. Hence, we discuss the pathogenesis and treatment methods of affected organs, including 2019 coronavirus disease (COVID‐19)‐related pneumonia, drowning, trauma, blood transfusion, severe acute pancreatitis, and sepsis. This review is intended to provide a new perspective concerning ARDS and offer novel insight into future therapeutic interventions.

## Graphical abstract

Acute brain injury (ABI), especially severe ABI, may cause dysfunction of peripheral extracranial organs and systems. “Blast injury” is one of the classic theories used to explain ABI‐related acute respiratory distress syndrome (ARDS). Sympathetic nervous system activation after brain injury changes the intravascular pressure, which damages the alveolocapillary membrane and causes neurogenic pulmonary edema. The management of ABI‐related ARDS patients requires multidisciplinary cooperation to ensure that patients receive the best treatment.

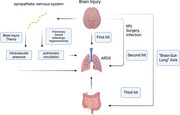

## 1 INTRODUCTION

Acute respiratory distress syndrome (ARDS) is a clinical syndrome of acute hypoxic respiratory failure caused by diffuse lung inflammation and edema. The mortality rate is high in ARDS patients and is related to the severity of the patient's condition.^1^ ARDS can be precipitated by intrapulmonary factors (pneumonia, aspiration, etc.) or extrapulmonary factors (severe acute pancreatitis [SAP], sepsis, trauma, etc.), which can lead to severe hypoxemia, decreased lung compliance, increased arteriovenous shunts, and increased physiological dead space. Patients suffering from ARDS have high mortality rates, including a 28‐day mortality rate of 34.8% and an overall in‐hospital mortality rate of 40.0%.^2^ Since ARDS was first described in 1967, its clinical definition has been revised several times.^3^, ^4^, ^5^ Each revision of the definition, in response to new research findings and clinical concepts, is intended to provide a definition that consistently and accurately identifies patients.

The pathophysiology of ARDS is complex and involves the activation and dysregulation of multiple overlapping and interacting pathways of systemic inflammation and coagulation, including the respiratory, circulatory, and immune systems. In general, the treatment of inflammatory injuries is a coordinated process that involves the downregulation of proinflammatory pathways and the upregulation of anti‐inflammatory pathways. Owing to differences in etiology and inducement, there is heterogeneity in the clinical syndrome. In recent years, foundation medicine, radiology, immunology, and other disciplines have increasingly recognized and incorporated phenotype recognition.

ARDS is a highly heterogeneous disease with different etiologies, different inflammatory phenotypes and different histomorphologic characteristics, which leads to relatively slow progress in the treatment of ARDS. Individualized medical approaches to the etiology of ARDS can improve the identification of ARDS phenotypes, which should contribute to a better understanding of its pathophysiological mechanisms and differences between patients. In this review, we discuss the current understanding of the structural features and changes in the lung function of ARDS patients. We further discuss the pathogenesis and treatment methods of affected organs according to their different causes.

## 2 ALVEOLAR STRUCTURE AND PHYSIOLOGICAL FUNCTIONS

**FIGURE 1:**
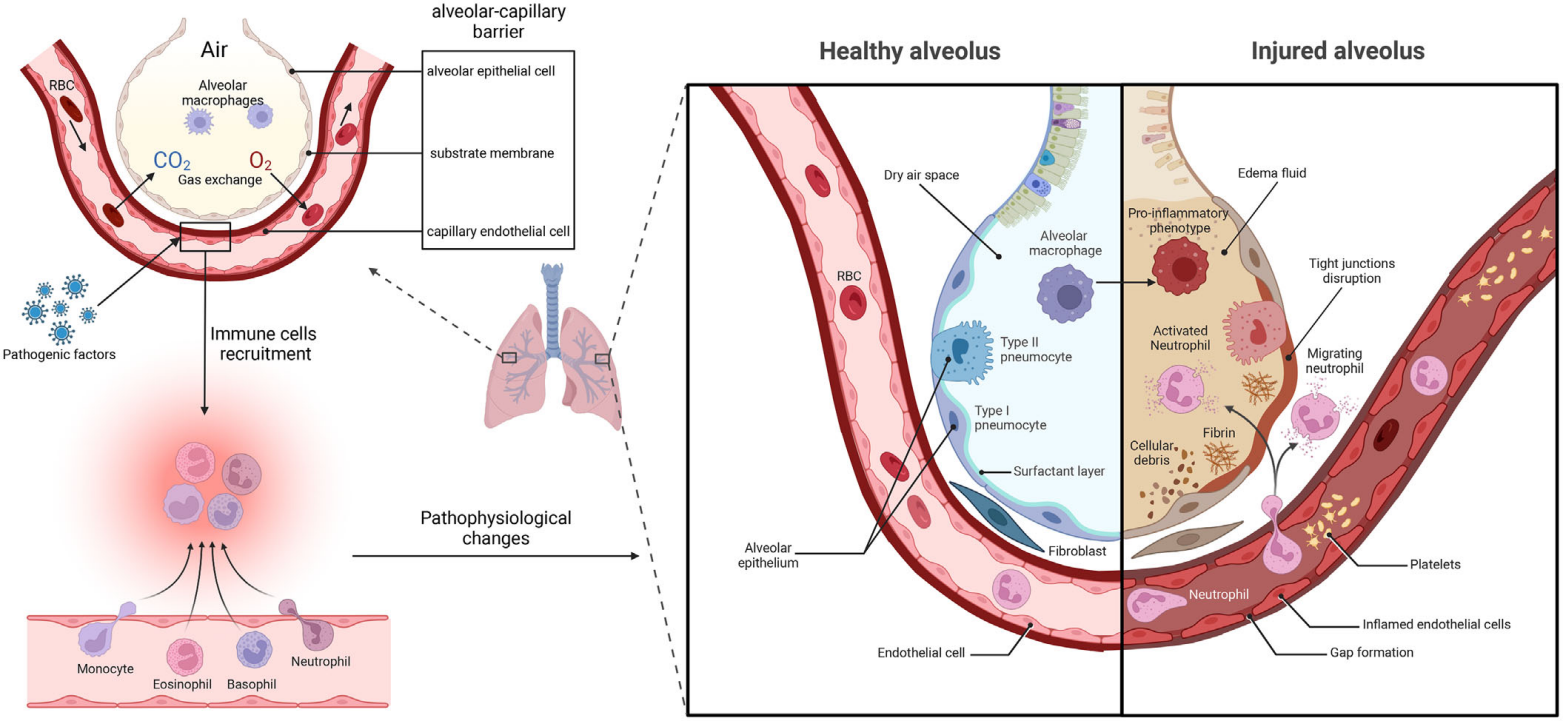
Alveolar structure and pathophysiological changes in acute respiratory distress syndrome (ARDS). The alveolar–capillary barrier is composed of alveolar epithelial cells (AECs) and capillary endothelial cells, which are separated only by a thin substrate membrane to facilitate gas exchange. Resident alveolar macrophages that fill the air space provide conventional defense, while many polymorphonuclear leukocytes (PMNs), including neutrophils, eosinophils, and monocytes, present in the alveolar capillaries can be quickly mobilized into the alveolar space in the event of infection. The typical characteristic of ARDS is damage to the alveolar–capillary barrier because fluid and protein pass through the alveolar epithelium due to increased permeability, leading to pulmonary interstitial edema.

The lungs are basically composed of bronchial trees formed by repeated branching of the bronchi. The characteristic of the lungs for gas exchange is the close relationship between the air space and capillaries. The bronchi repeatedly branch into the lungs, eventually forming alveoli around the terminal branches of this airway tree. In the walls of these alveoli, a dense capillary network connects to the terminal branches of the pulmonary arteries and veins.^6^ Therefore, the airway and blood vessels converge at a large and tightly connected interface. Through respiratory movement, air can be inhaled into bronchi and exchanged through the capillary network on the alveoli. Oxygen enters the blood from the air space while carbon dioxide in the blood enters the air space, achieving the balance of oxygen and carbon dioxide in the internal environment.^7^ The normal structure of lung facilitates the transfer of oxygen and the excretion of carbon dioxide in the alveolar–capillary unit (Figure 1).

The alveolar–capillary barrier is composed of alveolar epithelial cells (AECs) and capillary endothelial cells, which are separated only by a thin substrate membrane to facilitate gas exchange.^8^ The surface of the alveolar epithelium is lined with flat alveolar epithelial type I (ATI) cells and cuboidal alveolar epithelial type II (ATII) cells, the former of which allow gas exchange, whereas the latter produce surfactants to expand the lung under low surface tension. These two types of cells form a very tight barrier, limiting the passage of small solutes while allowing the diffusion of carbon dioxide and oxygen.^9^, ^10^

AECs express E‐cadherin and β‐catenin in the plasma membrane. As a transcriptional cofactor, β‐catenin plays a critical role in the renewal of ATII cells, which function as stem cells during homologous homeostasis. During the development of alveolar edema, the reabsorption of edema fluid depends on the connections between ATI and ATII cells, as well as the intact ion transport channels in epithelial cells, such as the apical sodium channel and the basal lateral Na^+^/K^+^‐ATPase pump.^9^, ^11^ Capillary endothelial cells function as regulatory factors in the process of fluid and inflammatory cell transfer into the interstitial space. These endothelial cells are connected by intercellular junctions, including tight junctions and adhesive junctions. The vascular endothelial cadherin (VE‐cadherin) contained in adhesive connections mediates intercellular contact through its extracellular domain and plays a critical role in barrier function.^12^

Normal alveoli are characterized by dry air areas, tight connections between epithelial and endothelial cells, a general level of surface active substances, standard Na–Cl transportation, normal passage of blood cells through capillaries, and the lack of expression of procoagulant factors or adhesion molecules. Generally, the fluid flowing out of capillaries carries water and low‐molecular‐weight solutes into the interstitial space through vascular walls and then into lymphatic vessels but cannot cross the epithelial barrier under normal circumstances. The natural cellular composition of alveoli includes alveolar macrophages (AMs), but not neutrophils, which can be rapidly recruited from the circulation. AMs, neutrophils, and other immunologic effector cells, including monocytes and platelets, are crucial in the regular defense mechanism of the lungs and play critical roles in acute lung injury.^13^ Resident AMs that fill the air space provide conventional defense, while many polymorphonuclear leukocytes (PMNs), including neutrophils, eosinophils, and mast cells present in the alveolar capillaries can be quickly mobilized into the alveolar space in the event of infection.^14^

## 3 PATHOPHYSIOLOGICAL CHANGES IN ALVEOLAR EPITHELIAL CELLS AND CAPILLARY ENDOTHELIAL CELLS IN ARDS

The typical characteristic of ARDS is damage to the alveolar–capillary barrier, which can be observed by electron microscopic analysis, directly leading to specific pathological and physiological manifestations. During the development of ARDS, the main alteration is the increased permeability of alveolar capillaries to fluid, protein, neutrophils, and other blood cells.^8^, ^10^, ^15^ Inflammatory dysregulation, inappropriate accumulation and activity of white blood cells and platelets, uncontrolled activation of coagulation pathways, and changes in the permeability of alveolar endothelial and epithelial barriers are the core pathological and physiological changes in acute lung injury and ARDS.^16^, ^17^ During ARDS, fluid and protein pass through the alveolar epithelium due to increased permeability, leading to pulmonary interstitial edema. Multiple factors can enhance the permeability of the alveolar epithelium and capillary endothelium, such as the disruption of VE‐cadherin homologous bonds.^18^ Owing to the impairment of the normally tight barrier properties of the alveolar epithelium, edema fluid is transferred to the alveoli, thereby damaging the transportation of fluid by the alveolar epithelium, which is an important mechanism for maintaining dry air space in alveoli.^10^, ^19^, ^20^ When severe edema develops in the alveoli, tight junctions are severely disrupted, leading to epithelial necrosis, transparent membrane formation, loss of sodium and chloride ion transport, peeling of glycosylated chemicals, increased levels of chemical factors and adhesion molecules, and the appearance of red blood cells in the airway.^20^

ARDS is caused by various direct causes, including pneumonia, inhalation lung injury, lung contusion, and chest trauma, as well as indirect causes, such as sepsis, shock, pancreatitis, cardiopulmonary bypass, and burns.^21^ Among ARDS patients, those with direct pulmonary causes have more alveolar epithelial damages and alveolar inflammation than those with indirect extrapulmonary causes.^22^ For example, in patients with direct pulmonary causes, the pathway dependent on caspase‐1 in the alveolar cavity is particularly upregulated, and when extrapulmonary risk factors lead to ARDS, endothelial damage and systemic inflammation increase. Since ARDS caused by extrapulmonary factors affects the entire lung through endothelial dysfunction, rather than more localized damage caused directly by the lungs, it is more common to lead to more extensive systemic damage.^23^, ^24^

The severity of lung epithelial injury varies in ARDS, from the activation of epithelial cells, the expression of adhesion molecules, the activation of proinflammatory and procoagulant pathways, and damages to intercellular connections, leading to a slight increase in paracellular permeability, to the significant necrosis of epithelial cells and detachment of the alveolar basement membrane.^25^ Owing to the necrosis of lung epithelial cells and leakage of intracellular contents, disease‐related molecules are released, which can amplify proinflammatory signals.^26^ The activation and damage of the alveolar epithelium can also lead to the shedding of anticoagulant molecules and the release of tissue factors from the lung epithelium into the alveolar space.^27^ Owing to the ability of lung epithelial cells to secrete antibacterial proteins, epithelial damage increases the risk of secondary infections as well.^28^

Early damage to ATI cells is usually severe and includes focal epithelial destruction and alveolar basement membrane detachment.^15^, ^29^ Epithelial cell necrosis usually occurs during the exudative phase, while the morphology of alveolar endothelial cells may be preserved.^30^ Following early damage to the alveolar epithelium, ATII cells rapidly proliferate,^10^, ^31^, ^32^ driving the release of procoagulant factors and fibrin deposition in the alveoli as well as near endothelial cells.^31^, ^32^, ^33^

Compared with severe damage to epithelial cells, the ultrastructural changes observed in alveolar endothelial cells during autopsy are often subtle.^10^ Experiments have demonstrated that endothelial cell activation can occur in ARDS, and the deposition of platelets and neutrophils is a result of endothelial cell activation, which typically manifests as neutrophil–platelet aggregation. Neutrophils and platelets seem to cooperate to increase the permeability of pulmonary blood vessels to proteins.^13^ Endothelial cell activation is usually induced by lung white blood cells and inflammatory signals.^34^ Moreover, damage to the vascular endothelium is more likely to increase systemic inflammatory reactions, which are caused by pathogens and toxins, barrier instability factors produced by AMs and circulating white blood cells, and various proinflammatory signaling molecules.^19^

## 4 ROLES OF MACROPHAGES, NEUTROPHILS, AND CYTOKINES IN ARDS

ARDS can be pathologically divided into the exudative phase, rehabilitation phase, and fibrotic phase.^25^ The pathophysiology of ARDS is very complex, and its mechanisms include the activation and dysregulation of multiple overlapping and interacting injury response pathways.^13^ Importantly, many of these pathways are normal responses to infection or injury, but excessive and diffuse activation is harmful.^13^, ^25^ A series of immune cells and cytokines play important roles in the development of ARDS.^35^ Immune cells, such as macrophages and eosinophils, play dual roles in ARDS development and disease prevention.

Many macrophages can be found in the alveoli of ARDS patients and exhibit significant phenotypic plasticity.^36^ To meet the functional requirements of different pathological phases in the microenvironment of ARDS, AMs can dynamically acquire a proinflammatory phenotype or an anti‐inflammatory/tissue remodeling phenotype.^37^ During the exudative phase of ARDS, macrophages are activated by pathogens through pattern recognition receptors (PRRs), including Toll‐like receptors (TLRs), leucine‐rich repeat sequence receptors (NLRs), transmembrane C‐type lectin receptors (CLRs), and retinoic acid‐induced gene‐like receptors (RLRs), which promote the transition of resident AM cells to the predominant proinflammatory phenotype.^38^ In the early stages of ARDS, activated macrophages secrete proinflammatory cytokines to clear pathogens, which may disrupt the structure of AECs and lead to cell death. In the late stage of ARDS, anti‐inflammatory cytokines secreted by selectively activated macrophages inhibit the inflammatory response, thereby promoting epithelial regeneration and alveolar structural remodeling.^14^ In addition to macrophages, a series of immune cells, including lymphocyte subsets and dendritic cells, as well as cytokine networks, jointly regulate alveolar inflammation in ARDS. In addition to pulmonary inflammation, ARDS patients generally suffer from systemic inflammation, which may lead to nonpulmonary organ failure in ARDS patients.^9^

Neutrophils are among the most important mediators of inflammation and lung tissue destruction during ARDS. Different interleukins assist in the activation of several signaling pathways, aiding in the secretion of other inflammatory or anti‐inflammatory interleukins and regulating the production and balance of immune cells involved in ARDS.^14^, ^35^ Neutrophil recruitment is accomplished primarily by macrophages that reside in and are recruited by the tissue, and lung epithelial cells can also release neutrophil chemotactic agents as well.^39^, ^40^ Macrophage PRRs bind to disease‐related or pathogen‐related molecules, activating macrophages into proinflammatory phenotypes and thereby triggering the release of proinflammatory cytokines and neutrophil chemoattractants such as interleukin (IL)‐8, tumor necrosis factor alfa (TNFα), and IL‐1β.^39^, ^41^ Neutrophils primarily enter the lungs through the capillary wall and move along the paracellular pathway between endothelial cells and AECs, which seems to be regulated by interstitial fibroblasts.^42^ During this process, multiple harmful mediators, including reactive oxygen species (ROS), proteases, and proinflammatory lipid mediators such as prostaglandins and leukotrienes, are released simultaneously.^43^

Transcription factor nuclear factor‐κB (NF‐κB) is one of the main regulatory factors in pulmonary cytokines induction.^44^, ^45^ A lack of NF‐κB subunit RelA reduces cytokine expression, neutrophils migration, as well as pulmonary bacterial killing.^46^ Cytokines are key factors in regulating pathways to guide, amplify, and resolve the immune response. However, excessive cytokines lead to the inflammatory cascade, resulting in epithelial and endothelial damages in ARDS. Proinflammatory cytokines, such as IL‐1β,^47^ IL‐8,^48^ IL‐6,^48^ and IL‐33,^49^ exacerbate lung injury in the acute phase of ARDS by activating inflammatory pathways. On the contrary, anti‐inflammatory cytokines, including IL‐10,^50^ IL‐22,^51^ IL‐13,^52^ and various chemokines,^53^, ^54^ exert protective effects at specific stages.

## 5 IMAGING CLINICAL MANIFESTATIONS

Radiological examinations for ARDS mainly include chest X‐ray and chest computed tomography (CT) examinations, which can be used as auxiliary tools for the diagnosis and evaluation of ARDS.^55^ On the one hand, the etiology of ARDS, such as the cause of direct lung injury, can be inferred, including different types of pneumonia, pulmonary embolism, aspiration, and pulmonary hemorrhage. Radiological examination reveals visual lung changes, such as massive inflammation, exudation, pneumothorax, lung contusion, intrapleural hemorrhage, rib fracture. On the other hand, radiology studies can assist in the clinical evaluation of different pathological stages of the disease. For example, in the stage of exudation and early proliferation, scattered patchy ground–glass opacity can be observed in both lungs, accompanied by interlobular septum thickening and reticular changes. Mild dilation of the peripheral bronchioles can be used as an indication of the early stage of proliferation. As the disease progresses, in the late stage of proliferation, traction bronchial wall thickening, mild lumen dilation, and decreased lung volume (transposition of interlobar vessels and bronchi) may be observed. By the time fibrosis occurs, in addition to traction bronchiectasis, large grid‐like and small cystic changes may be observed in the lungs. To further quantify the pathological changes associated with pneumonia, the AI‐assisted analysis of the CT images has been applied to quantify the fibrosis.^56^ In contrast to conventional CT, molecular imaging can noninvasively provide spatial resolution information about the pathological processes that lead to ARDS. Philip developed a positron emission tomography imaging agent that detects pulmonary inflammation during acute lung injury. The quantification of radiotracer uptake strongly correlated with the expression of established inflammatory markers. After treatment with dexamethasone to reduce inflammation, minimal tracer uptake in the consolidation zone was confirmed, highlighting the potential to quantify the burden of consolidation pneumonia.^57^ Additionally, ^99m^Tc‐HMPAO and ^99m^Tc‐duramycin have been used to detect pulmonary oxidative stress and cell death, which has the potential utility of identifying high‐risk hosts that are susceptible to developing ARDS.^58^ Thus, different etiologies and pathologies of ARDS reflect different imaging manifestations, and clinicians need to adjust the treatment plan by combining various clinical manifestations.

## 6 COVID‐19‐RELATED PNEUMONIA

Severe acute respiratory syndrome coronavirus type 2 (SARS‐CoV‐2), which causes 2019 coronavirus disease (COVID‐19), has been proven to lead to a series of serious consequences, including ARDS, systemic inflammation, multiple organ failure, and death.^59^ Research on COVID‐19 has demonstrated that there are many physiological similarities between COVID‐19‐related ARDS and traditional ARDS.^60^ Eckermann et al.^61^ analyzed the lung samples of six patients who died of respiratory failure due to COVID‐19 at different times after SARS‐CoV‐2 infection. Analysis revealed typical manifestations of diffuse alveolar damage, including the formation of a transparent membrane near AECs, with moderate lymphocyte infiltration and a single thrombus in small pulmonary veins. In addition, many swollen and inflamed blood vessels and thick transparent membrane samples were found. The pulmonary capillaries were filled with congested red blood cells in all the samples.

A retrospective study by Brault et al.^62^ included 63 patients with moderate to severe primary ARDS, 24 (38%) of whom were diagnosed with SARS‐CoV‐2 infection and 39 (62%) of whom developed ARDS due to other traditional causes. The prevalence of immunodeficiency in the non‐COVID‐19 group was significantly greater (*p* = 0.004). The median time (interquartile range [IQR]) between symptom onset and orotracheal intubation was longer in the COVID‐19 group (10 days vs. 5 days; *p* = 0.0001). This study revealed that the pulmonary mechanics of COVID‐19‐related ARDS were relatively unaffected, but the degree of gas exchange was greater than that of traditional ARDS patients. The dissociation between pulmonary mechanics and gas exchange was not greater in the COVID‐19 group than in the non‐COVID‐19 group. Diffuse basal layer opacification is more common in COVID‐19 ARDS patients, whereas pleural effusion is less common. However, there are several different characteristics between COVID‐19‐related ARDS and traditional ARDS, including delayed onset, malignant inflammatory response, and pulmonary microthrombi.^63^ Early reports indicated a high incidence rate of venous thrombosis and coagulation disorders in patients with COVID‐19‐related ARDS.^64^ The levels of IL‐6, TNF‐α, and IL‐8 are reportedly significantly increased in the serum of patients with COVID‐19. In particular, the initial levels of IL‐6 and IL‐8 are closely related to the severity of COVID‐19, which manifests as pulmonary imaging disasters, reduced creatinine clearance, the need for vasopressors and mechanical ventilation (MV), and even further development into ARDS.^65^ A multicenter prospective observational study including 301 patients with COVID‐19 revealed that the lung morphology and respiratory mechanics of ARDS patients with COVID‐19 are generally consistent with those of classical ARDS patients. Moreover, the mortality is significantly greater in a subgroup of patients with COVID‐19‐related ARDS characterized by low static compliance of the respiratory system and high concentrations of D‐dimer than in other patients.^66^

Despite the specificities of COVID‐19‐related ARDS, there are several similarities with COVID‐19‐related ARDS and traditional ARDS treatments; for example, lung‐protective ventilation and prone positioning should be widely used.^63^ The efficacy of steroids in the treatment of COVID‐19 and the necessity of systemic anticoagulation have been confirmed. The RECOVERY trial is a large practical randomized open study conducted in the United Kingdom, which suggested that the application of dexamethasone 6 mg/day for 10 days was associated with a lower mortality rate of hospitalized patients with COVID‐19 within 28 days, and the effect was most obvious in patients receiving MV.^67^ A prospective meta‐analysis including seven randomized trials and 1703 patients reported that the overall odds ratio (OR) of death from different steroid treatments was 0.66 (95% confidence interval [CI], 0.53–0.82, *p* < 0.001). Compared with conventional treatment or placebo (three trials, 1282 patients and 527 deaths included), the fixed effects summary OR of dexamethasone related to mortality was 0.64 (95% CI, 0.50–0.82; *p* < 001), the OR of hydrocortisone was 0.69 (95% CI, 0.43–1.12; *p* = 0.13), and the OR of methylprednisolone was 0.91 (95% CI, 0.29–2.87; *p* = 0.87).^68^

On the basis of the specificity of COVID‐19, several novel treatment options are being explored. A retrospective cohort study^69^ revealed that initiating continuous positive airway pressure (CPAP) therapy is beneficial for treating COVID‐19‐related ARDS patients compared with high‐flow nasal cannula (HFNC) therapy. The incidence of intubation in the CPAP group was significantly lower than that in the HFNC group, and the survival rate after 60 days was remarkably improved. A randomized controlled trial revealed that treatment with mesenchymal stromal cells (MSCs) did not reduce mortality within 30 days or improve mortality without ventilation within 60 days after randomization in patients with moderate‐to‐severe COVID‐19‐associated ARDS.^70^ Babajani et al.^71^ found that human amniotic epithelial cells (hAECs) and their secretions have therapeutic effects on COVID‐19 by regulating immune system responses and controlling coagulation disorders. Moreover, hAECs have potential benefit in preventing pulmonary fibrosis, clearing alveolar fluid, and participating in tissue regeneration in clinical and preclinical models of ARDS. Preconditioning and gene modification can enhance the therapeutic effect of hAECs.

In recent years, vaccines against COVID‐19 have been developed, and more than 10 billion doses have been administered worldwide. The adverse events related to the COVID‐19 vaccine for severe acute respiratory syndrome have recently attracted attention. Myocarditis, thrombocytopenia, and thromboembolism are rare but well‐known side effects, whereas vaccine‐induced ARDS is not common.^72^, ^73^ Kawano et al. reported a case^74^ in which a patient developed ARDS after receiving the SARS‐CoV‐2 vaccine, and the autopsy results confirmed this diagnosis. Despite intensive care, including corticosteroids and extracorporeal membrane oxygenation (ECMO), the patient did not survive. However, the exact mechanism of the serious side effects caused by vaccines remains unclear, with abnormal immune responses possibly involved. The pneumonia virus can mutate very quickly, the response of patients in different regions is also very different, and the treatment of the disease caused by it needs to adapt.

## 7 DROWNING

**FIGURE 2:**
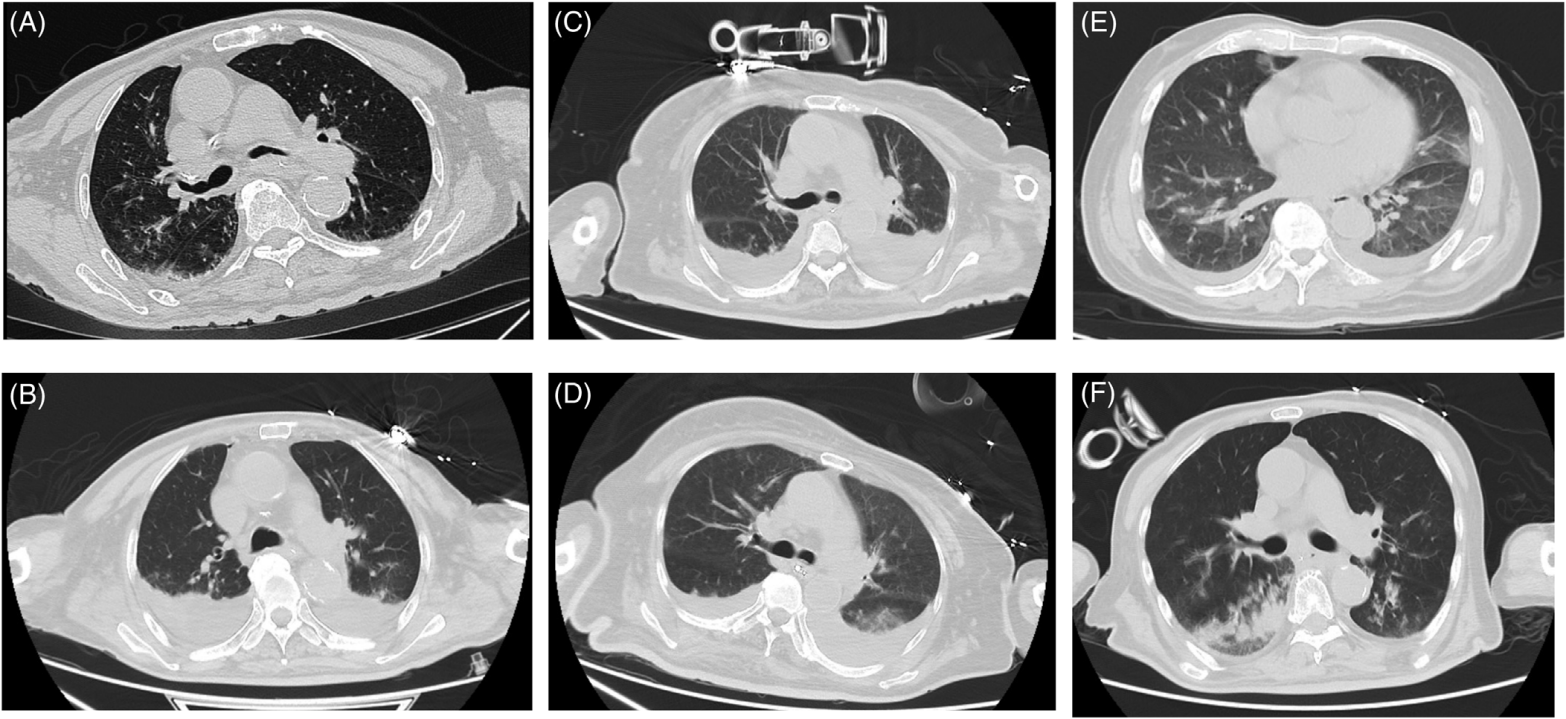
Trauma‐associated acute respiratory distress syndrome (ARDS). (A, B) A 78‐year‐old man with cerebral infarction developed dyspnea after mistakenly absorbing water and decreased finger pulse oxygen. An emergency computed tomography (CT) revealed bilateral pneumonia (A). After 7 days CT revealed scattered inflammation in both lungs and increased pleural effusion (B). (C, D) A 69‐year‐old female presented with dyspnea and 88% finger pulse oxygen 1 month after head injury (subdural hematoma). CT revealed bilateral pleural effusion (C). CT revealed that degree of pleural effusion decreased after fecal transplantation (D). (E) A 63‐year‐old male presented with dyspnea and low blood oxygen 3 days after abdominal surgery (laparoscopic radical resection of rectal cancer). CT showed bilateral pulmonary exudation. (F) An 80‐year‐old male with kidney injury caused by car accident. After strictly bed rest 6 days, CT revealed a few scattered bands shadows in both lungs and inflammation in the lower lobe of the right lung.

The World Health Organization defines drowning as “the process of respiratory difficulty caused by submersion/immersion in liquid.”^75^ Drowning is responsible for more than 40 deaths every hour.^75^ Cough is the first reflex when liquid enters the respiratory tract, which involves the inhalation of more liquid.^75^ These fluids can directly prevent oxygen exchange and cause hypoxemia. The water in the alveoli causes surfactant destruction and washout, initiating acute lung injury (Figure 2A,B).^76^ In addition, changes in osmotic pressure destroy the alveolar–capillary membrane and aggravate lung injury.^76^, ^77^ Without timely rescue, respiratory and circulatory failure can lead to death. Cardiopulmonary resuscitation (CPR) is often necessary depending on the severity of drowning (checks of consciousness, breathing, pulse, etc.). CPR administered continuously until signs of life appear or when rescuers provide basic/advanced life support.^76^, ^77^ Due to the low incidence of ventricular fibrillation or ventricular tachycardia, automated external defibrillators (AEDs) have a limited effect in rescue drowning people. Treatment in hospitals should consider the severity of the patient's condition according to a comprehensive examination, including assessments of circulation, respiration, and the nervous system. MV with positive end‐expiratory pressure (PEEP) should be used for patients with hypoxemia to ensure an intrapulmonary shunt (QS: QT) of ≤20% or PaO_2_:FiO_2_ of ≥250.^76^ Antibiotics should be used to prevent pneumonia, whereas corticosteroids should not be used except for bronchospasm.^76^ It is also important to adjust the circulation capacity and electrolyte and acid–base balance. The outcome of drowning patients varies from nearly no physical damage to death depending on the severity of the drowning. Thus, prevention is the foremost issue. However, when drowning occurs, timely rescue and treatment is the key to reduce mortality, in which delivery of oxygen and perfusion of tissues are particularly important.

## 8 TRAUMA‐ASSOCIATED ACUTE RESPIRATORY DISTRESS SYNDROME

While sepsis is the leading risk factor for ARDS, trauma is also a major risk factor for ARDS. Owing to the high degree of uncertainty associated with trauma, the incidence of trauma‐induced ARDS is not well established. Previous statistics have demonstrated that trauma contributes to approximately 6% of ARDS cases.^78^ Although the mortality rate is also lower than that of infective ARDS, the mortality rate is approximately about 24%.^79^, ^80^ Many types of trauma in the human body, including brain injury, lung injury, gastrointestinal injury, and fracture, lead to ARDS through different pathophysiological processes. The treatment also varies according to their pathophysiological processes. Clinicians may need to constantly adjust treatment strategies on the basis of dynamic changes in organ‐specific physiology, but there are also several some common features, such as lung‐protective ventilation.

### 8.1 Acute brain injury

Acute brain injury (ABI), especially severe ABI, may cause dysfunction of peripheral extracranial organs and systems. The lung might be the most vulnerable site to damage after ABI. Many studies have demonstrated that ARDS and the ABI have a bidirectional effect, predicting a worse outcome.^81^, ^82^, ^83^ However, the pathophysiology between the brain and lung is complex and includes inflammation, immune suppression, and neurodegeneration.^84^, ^85^

**FIGURE 3:**
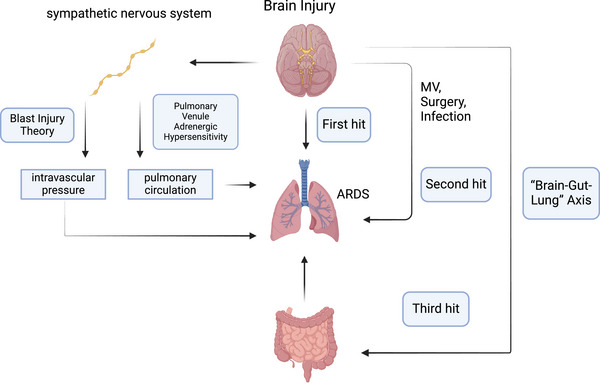
Pathophysiology of acute brain injury‐associated acute respiratory distress syndrome (ARDS). “Blast injury” theory, “pulmonary venule adrenergic hypersensitivity” theory, “double‐hit” theory, and “triple‐hit” theory. MV, mechanical ventilation.

“Blast injury” is one of the classic theories used to explain ABI‐related ARDS. Sympathetic nervous system activation after brain injury changes the intravascular pressure, which damages the alveolocapillary membrane and causes neurogenic pulmonary edema (Figure 3).^86^, ^87^, ^88^ “Pulmonary venule adrenergic hypersensitivity” suggests that the ABI may overactivate the sympathetic nervous system, and release large amounts of catecholamine hormones such as epinephrine and norepinephrine. These hormones can cause strong contraction of pulmonary venules, increasing the resistance of the pulmonary circulation, which may cause ARDS.^87^, ^89^ The “double‐hit” theory was proposed in the last decade to explain the inflammatory response in ABI‐related ARDS.^90^ Many inflammatory mediators are produced in the ABI, enter the circulation, and create a proinflammatory environment (first hit). The “first hit” makes the extracranial organs more susceptible to other inflammation‐inducing factors (such as MV, surgery, and infection), which constitute the “second hit.”^91^, ^92^ In recent years, the popular theory “triple‐hit” has been proposed on the basis of the “double‐hit” theory, which adds the “brain–gut–lung” axis.^93^ Many studies have demonstrated that ABI can affect the motility and permeability of the gastrointestinal tract and alter the composition of the gut flora, leading to dysregulation of the gut flora. These disturbed changes in intestinal function and microorganisms have adverse effects on the defense of the lungs against pathogens and constitute the “third hit” (Figure 3).^94^, ^95^, ^96^, ^97^

The treatment of ABI‐induced ARDS is very difficult because of various pathophysiological changes. Many clinical studies have demonstrated that MV, low tidal volume and high PEEP can significantly improve the prognosis of ARDS patients.^98^, ^99^ However, long‐term low tidal volume ventilation may induce hypercapnia, which leads to vasodilation of cerebral arterioles with subsequent intracranial hypertension and brain tissue hypoxia.^84^ The application of high PEEP increases intrathoracic pressure, which affects venous return to the brain and ultimately leads to increased intracranial pressure (ICP).^83^ This worsens the ABI. Therefore, it is necessary to balance the contradiction between lung ventilation and brain perfusion. Recent recommendations for ventilation targets in patients with ABI with ARDS are that PaO_2_ levels should be maintained between 80 and 120 mm Hg.^100^ Some studies suggest that the effect of PEEP on ICP may be small if the PaCO_2_ can be reasonably controlled. However, high PEEP levels should be avoided.^82^, ^84^ However, considering individual differences, MV parameters need to be adjusted individually to maintain cerebral perfusion and the ICP.

The prone position is a readily implementable intervention associated with improved oxygenation due to an increased ventilator–blood flow ratio, more uniform ventilation distribution, increased lung volume, and improved perfusion redistribution during partial alveolar re‐expansion in the respiratory‐dependent area of the lung.^101^, ^102^ In the prone position, the effects of cardiac, gravity, and chest wall compression on the supine‐dependent portion of the lungs are reduced.

In the treatment of ARDS patients, the prone position can improve oxygenation and reduce mortality.^103^, ^104^ The prone position can decrease overdistension in nondependent lung regions and cyclical opening and closing in dependent lung regions, which decreases mortality in patients with ARDS.^105^, ^106^ During the COVID‐19 pandemic, many studies demonstrated that prone ventilation can improve the prognosis of ARDS patients.^107^, ^108^ However, an incorrect prone position can increase intrathoracic pressure via diaphragm elevation, causing impaired blood outflow from the brain and leading to an increase in the ICP.^109^ However, if the ICP is well controlled, the prone position is strongly recommended for patients with ABI‐induced ARDS.^100^

Gut microbiota regulation is also one of the current treatments for ABI‐related ARDS on the basis of the “brain–gut–lung” axis (triple‐hit theory). Probiotics can significantly alter the pathogenesis of inflammatory processes by promoting the proliferation of healthy beneficial microorganisms and reducing the presence of pathogenic microorganisms.^94^ This regulatory effect affects the development and maturation of the innate and adaptive immune systems by influencing the secretion of cytokines, such as IL‐10 and transforming growth factor (TGF)‐β.^110^ Fecal microbiota transplantation is also a promising treatment (Figure 2C,D). Both animal studies and small‐scale clinical trials have demonstrated that ABI patients receiving fecal transplants help restore the normal gut microbiota and exhibit additional neuroprotective effects.^111^, ^112^, ^113^

ECMO directly provides conditional support for patients, improves the blood oxygen exchange rate, and has an important impact on the adjustment of body hemodynamics. In the past, brain injury with severe ARDS was regarded as a contraindication for ECMO, as the use of ECMO may aggravate ABI.^114^ However, in recent years, there have been attempts to extend the lives of some patients.^115^, ^116^ There may be more adjustments in the future to resolve conflicts in the current treatment.

The management of ABI‐related ARDS patients requires multidisciplinary cooperation, including neurology, respiratory, and intensive care unit (ICU) cooperation, to ensure that patients receive the best treatment. Preventive measures include control of ICP, hemodynamic stability, and appropriate MV strategies. In addition, monitoring the lung condition of patients with brain injury and timely detection and management of lung complications are essential to improve the prognosis of patients.

### 8.2 Traumatic thoracic injuries

Traumatic thoracic injury is caused mainly by traffic accidents, falls and crush injuries and causes significant morbidity and trauma‐related death, including osseous, pneumonic, heart and vessel, and diaphragm injuries. Traumatic thoracic injury can directly affect lung ventilation function, leading to ARDS. These injuries damage the alveolocapillary membrane, leading to alveolar edema, surfactant destruction and hypoxemia. Pain caused by trauma also has a significant impact on ventilation function.^117^, ^118^

Emergency medical measures should be activated in a timely manner, aiming to restore ventilation function and reduce complications. This includes treating injuries, maintaining unobstructed breathing, administering oxygen, controlling bleeding, replenishing blood volume, and providing analgesia. These treatments need to be adjusted according to the patient's injury. For patients with severe chest injuries, such as tension pneumothorax and hemothorax, emergency surgery needs to be performed immediately. MV with a low tidal volume (approximately 6 mL/kg), low plateau pressure (30 cm of water or less), and increased respiratory rate might decrease mortality.^119^, ^120^ A recent randomized controlled trial revealed that, compared with conventional oxygen therapy and late non‐invasive ventilation (NIV), the combination of HFNC‐O_2_ with preventive NIV did not reduce the rate of endotracheal intubation or secondary respiratory complications in high‐risk blunt chest trauma patients with nonsevere hypoxemia and no sign of acute respiratory failure.^121^

The prognosis of ARDS patients is affected by many factors, such as age, health status, and severity of injury. In summary, timely identification and multidisciplinary comprehensive treatment are the keys to improving patient prognosis.

### 8.3 Abdominal trauma

**FIGURE 4:**
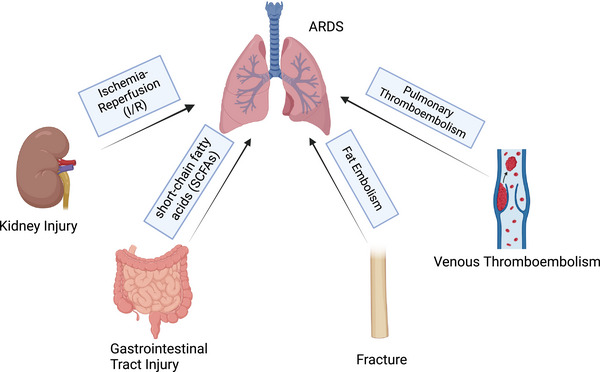
Pathophysiology of trauma‐associated acute respiratory distress syndrome (ARDS). “Kidney–lung axis,” “gut–lung axis,” fractures, and venous thromboembolism.

Owing to the numerous organs in the abdominal cavity, injury may lead to simultaneous damage to multiple systems, including the gastrointestinal tract and urinary tract. Thus, evaluating abdominal trauma is a challenging, and the pathological and physiological changes vary significantly depending on the type of injury. ARDS is also a common complication after abdominal injury. ARDS is usually associated with a systemic inflammatory response after abdominal injury, increased abdominal pressure, diaphragmatic dysfunction, immune regulation, abdominal surgery, hemorrhagic shock, fluid resuscitation, and other factors.^122^ Gastrointestinal tract injury can lead to ARDS through the “gut–lung axis,” as we mentioned in the ABI chapter.^96^ The gut microbiota produces short‐chain fatty acids (SCFAs) through anaerobic fermentation of dietary fiber and resistant starch.^123^, ^124^, ^125^ SCFAs are involved in many pathophysiological processes, including immune cell migration, AM polarization, and the regulation of neutrophil extracellular trap (NET) formation in the lungs, and lead to lung injury or ARDS (Figures 2E and 4).^123^, ^126^, ^127^ Acute kidney injury can also cause lung injury through the “kidney–lung axis.”^128^ Some studies have confirmed that renal ischemia–reperfusion (I/R) injury may lead to mitochondrial dysfunction, which may cause ARDS (Figures 2F and 4).^129^, ^130^ The “liver–lung axis” also affects the microbiome composition, leading to immune dysregulation, promoting chronic inflammation, and affecting the lung.^131^, ^132^

Like traumatic thoracic injuries, emergency medical measures should be activated in a timely manner. Abdominal trauma‐related ARDS is a serious clinical problem, and it is necessary to consider the management of abdominal injury and the treatment of ARDS. Multidisciplinary management, including fluid management, MV, and the management of abdominal injuries is usually needed.

### 8.4 Fracture

Fractures are common injuries that often occur in car accidents, falls, or violent injuries. Fractures can cause direct damage to lung tissue (such as ribs in traumatic thoracic injury), systemic inflammation and infection, fat embolism, and thrombus due to long‐term immobilization, leading to lung injury and ARDS.^133^, ^134^ Intramedullary fat can be disrupted in long bone fractures and orthopedic surgery, which can cause fat embolism syndrome (FES). FES usually manifests as changes in the lungs, central nervous system and skin, and respiratory symptoms are the most common. After fracture, the intramedullary fat enters the blood and circulation. Fat can obstruct pulmonary capillaries directly and lead to a ventilation–perfusion mismatch, low oxygen saturation, and dyspnea.^135^ In addition, the blockage of blood vessels caused by fat droplets can cause inflammation, leading to endothelial damage, permeability edema, and eventually ARDS (Figure 4).^135^ At present, there is no gold standard for FES diagnosis. FES is usually diagnosed by medical history, disease changes, and imaging examinations. The most common feature on high‐resolution computed tomography (HRCT) is patchy ground–glass opacities, which are often associated with smooth interlobular septal thickening.^135^, ^136^ A small portion manifests as small centrilobular nodules in subpleural regions.^137^ The current treatment options are mainly symptomatic and supportive. During treatment, prevention, monitoring, and early detection are very important.

**FIGURE 5:**
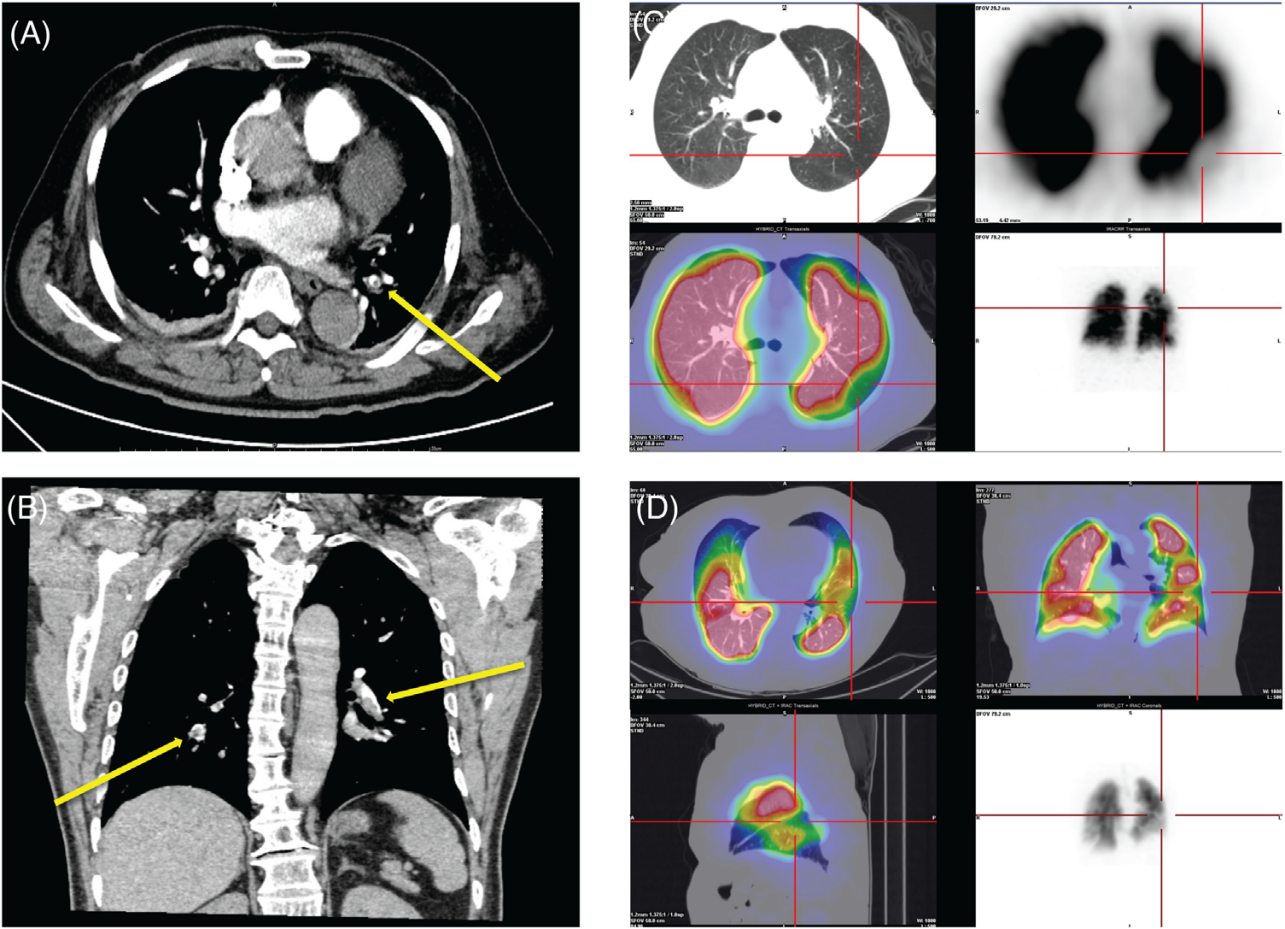
Pulmonary embolism after trauma (computed tomography pulmonary angiogram [CTPA] and pulmonary perfusion imaging). (A) A 66‐year‐old male presented with asymptomatic hypoxia and high D‐dimer levels (2.1 µg/mL; reference value 0–0.55 µg/mL) 4 days after abdominal surgery (laparoscopic radical prostatectomy). CTPA revealed pulmonary embolism in the left lower lobe. (B) A 68‐year‐old male presented with dyspnea 3 days after kidney injury. The D‐dimer concentration was 6.23 µg/mL. CTPA revealed that multiple emboli had formed in the anterior and posterior segments of the left upper lobe and pulmonary artery branches of the lower lobes. (C) An 81‐year‐old male presented with dyspnea 5 days after femoral fracture. Pulmonary perfusion imaging revealed a pulmonary embolism in the left upper lobe. (D) An 80‐year‐old female with an epidural hematoma caused by a fall injury developed a pulse oxygen level of 90% (with air) 7 days later. Pulmonary perfusion imaging revealed a left pulmonary embolism.

Venous thromboembolism (VTE) is a fatal complication after trauma and surgery. Due to limited activity, VTE events after fracture are not uncommon.^138^ The pathophysiological process of VTE is complex and includes venous flow, the endothelium, platelets, leukocytes, and the interaction between inflammation and hemostasis (Figure 4).^139^ When the formed thrombus is detaches and spreads to the lungs with blood flow, it blocks the pulmonary arteries, causing dyspnea, hypoxemia, and even sudden death. The treatment of VTE focuses on prevention. At present, many clinical scales, such as the Caprini scale and the Padua scale, are used to assess the probability of VTE events. The preventive measures include mechanical prophylaxis (stretch socks, inflatable compression pumps, and early activity) and pharmaceutical prophylaxis (anticoagulants). The D–D dimer is a commonly used clinical indicator for VTE evaluation; other indicators, including activated partial thromboplastin time, fibrinogen, thrombin generation, C‐reactive protein, and the erythrocyte sedimentation rate, also have reference value.^140^ Once patients present with clinical manifestations such as hypoxemia and high DD dimer, the diagnosis of pulmonary embolism needs to be considered. CT pulmonary angiogram (CTPA) or pulmonary perfusion imaging is currently a specific test for the diagnosis of pulmonary embolism (Figure 5).^141^ Treatment for pulmonary embolism includes supportive therapy (maintenance of respiratory circulation), anticoagulant therapy (low‐molecular‐weight heparin and direct oral anticoagulant drugs), thrombolytic therapy, and interventional or surgical thrombectomy.^142^, ^143^

ARDS can be triggered by a diverse array of injuries and pathophysiological processes. Therefore, it is imperative to meticulously assess the severity of the injury and continuously monitor the patient's clinical status. Injury is not solely a localized phenomenon, its diagnosis and management necessitate a comprehensive and holistic approach that takes into account the entire body.

## 9 BLOOD TRANSFUSION‐RELATED ACUTE LUNG INJURY

Blood transfusion is an important means of clinical treatment that can maintain the circulating blood volume and oxygen‐carrying capacity of the patient, improve hemostatic function, correct anemia, and save the patient's life. As a special medicine, blood saves the life of patients but also inevitably has the risk of adverse transfusion reactions, threatening the health of patients and increasing the medical burden. In recent years, increasing attention has been given to the safety and adverse reactions associated with blood transfusions worldwide.

Transfusion‐related acute lung injury (TRALI) refers to sudden acute dyspnea that occurs during or within 6 h after transfusion. Acute noncardiogenic pulmonary edema and hypoxemia are the main clinical manifestations, which is one of the primary causes of transfusion‐related death and seriously threatens the safety of transfusion.^144^

The pathophysiological mechanism of TRALI is relatively complex, and its incidence and development involve a series of biological processes, such as inflammatory cell infiltration, oxidative stress, alveolar–capillary barrier destruction, permeability changes, and cell apoptosis.^145^, ^146^, ^147^ Many immune‐ and inflammation‐related signaling molecules and signaling pathways are involved. The “double‐hit” theory is a hypothesis that suggests that TRALI may occur when a susceptible individual experiences the first hit, such as trauma, surgery, smoking, alcoholism, MV or sepsis, and then receives a blood product (the second hit), which contains antibodies, inflammatory mediators, and cytokines.^144^, ^148^

The diagnosis of TRALI is as follows: the patient's symptoms occur during or within 6 h after transfusion, and the following conditions are met: (1) the patient has no acute lung injury before blood transfusion; (2) sudden acute hypoxemia, with an oxygenation index under natural respiration (PaO_2_/FiO_2_) ≤300 mm Hg or blood oxygen saturation (SpO_2_) < 90% or other clinical manifestations of hypoxemia; and (3) imaging examination clearly reveals bilateral pulmonary edema; and (4) no or combined left atrial hypertension that is not the main cause of hypoxemia. (*) However, patients with a high risk for ARDS or mild symptoms of ARDS, whose respiratory status is stable within 12 h prior to transfusion and whose respiratory status deteriorates because of transfusion should also be considered for TRALI. In 2019, TRALI was categorized into two types: type I (without ARDS risk factors) and type II (with ARDS risk factors or mild ARDS). However, TRALI remains an exclusionary diagnosis, and there are no diagnostic tests or specific diagnostic markers available. In addition, there is no effective clinical treatment. Blood transfusions should be stopped immediately when TRALI is suspected. The main clinical treatment is MV and other symptomatic support treatments. Some studies have been conducted to regulate biological processes such as inflammation, immunity, and blood clotting, aiming to improve the treatment and prognosis of TRALI.^148^ However, more experimental and clinical studies are needed in the future.

## 10 IMMUNE CHECKPOINT INHIBITOR PNEUMONITIS

Immune checkpoint inhibitors (ICIs) have made significant progress in the treatment of tumors, but they may also cause immune‐related adverse events (irAEs), including ICI pneumonitis, which can lead to ARDS.^149^, ^150^, ^151^ ICIs enhance the body's antitumor immune response by blocking inhibitory signaling pathways, such as PD‐1, PD‐L1, and CTLA4, on T cells. However, this immune activation can be overdone, leading to an attack on normal tissues, including skin, gastrointestinal tract, liver, and lungs.^152^, ^153^ Skin toxicity usually occurs after 2–3 weeks of medication, while adverse reactions to the gastrointestinal tract (after 5 weeks) and liver and lung (after 6–7 weeks) occur at a later time. Checkpoint inhibitor‐associated pneumonitis (CIP) incidence in clinical trials was reported to be <6%, but real‐world data revealed a higher rate ranging between 10% and 20%.^154^ The clinical symptoms include dyspnea, cough, fever, and chest pain. Imaging examinations reveal ground glass nodules or patchy shadows,^155^, ^156^ which need to be differentiated from lung infections or lung tumors. Some studies have noted that ICIs are involved in physiological processes such as inflammation and apoptosis, which lead to ARDS.^157^

The American Society of Clinical Oncology Clinical Practice Guidelines (ASCO Guidelines) classify ICIs pulmonary toxicity into four levels on the basis of symptoms and examination results. G1: Asymptomatic, confined to one lobe of the lung or <25% of the lung parenchyma, clinical or diagnostic observations only. G2: Symptomatic, involving more than one lobe of the lung or 25%–50% of the lung parenchyma, medical intervention indicated, limiting instrumental activities of daily living (ADL). G3: Severe symptoms, hospitalization needed, involvement of all lung lobes or >50% of the lung parenchyma, limiting self‐care ADLs, with oxygen therapy indicated. G4: Life‐threatening respiratory compromise, urgent intervention indicated (intubation).^158^, ^159^ Other guidelines have similar grading methods, such as the Chinese Society of Clinical Oncology(CSCO) guidelines.^160^

Treatment may include the discontinuation of immunotherapy, the administration of corticosteroids and other immunosuppressants, and supportive care, such as oxygen therapy and MV.^161^ Glucocorticoids such as prednisone (1–2 mg/kg/day) remain the primary modality for treatment, and it is advisable to adhere to the principle of gradual tapering. Concurrently, preventive measures against related complications should include antibiotics, calcium supplements, proton pump inhibitors (PPIs), and other relevant treatments.^150^, ^162^, ^163^ However, if there is no improvement within 48 h under glucocorticoid therapy, the combined use of immunosuppressants should be considered, such as infliximab 5 mg/kg or mycophenolate mofetil IV 1 g twice a day, intravenous immunoglobulin for 5 days or cyclophosphamide, should be considered. Corticosteroids are used for more than 4–6 weeks.^158^ However, the safety of immunosuppressants in managing irAEs remains contentious, as immunosuppressants may predispose patients to infections, tumor progression, and exacerbation of their condition.^164^ ICIs may resume when patients are in G1 with radiographic evidence of improvement or resolution.^158^ The treatment of ICI‐related ARDS remains a challenge, and more research is needed to optimize management strategies. In addition, patients receiving ICIs should be closely monitored to detect and manage possible pulmonary complications in a timely manner.

## 11 ACUTE PANCREATITIS

**FIGURE 6:**
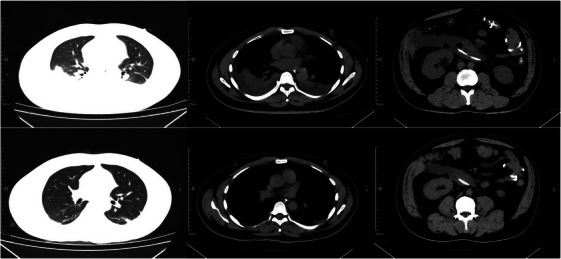
Systemic computed tomography (CT) imaging changes in severe acute pancreatitis (SAP). Imaging changes before and after treatment in a 37‐year‐old male patient with SAP (biliary tract stones, hyperlipidemia).

Acute pancreatitis (AP) is an acute abdominal disease caused by pancreatic enzyme activation through a variety of triggers. Biliary diseases, hyperlipidemia and alcohol use were assessed as the causes of AP (Figure 6). Additionally, autoimmune disease and *Leptospira* infection are contributing factors.^165^, ^166^ Owing to its various clinical features, and potential to progress to SAP, this condition is associated with high mortality and a poor prognosis.^167^, ^168^, ^169^ In terms of the Frailty Risk Score, patients vary widely in severity. The rates of respiratory complications, including pleural effusion, acute respiratory failure, and ARDS (0.22% vs. 0.95%), are high in frail patients.^170^ AP‐related acute lung injury (ALI) can progress to ARDS, which accounts for 60% of all deaths within the first week.^171^ Moreover, the lung is considered the initial distal organ affected by AP,^172^, ^173^ and some studies have been proposed to explain the pathophysiology of AP and its related pulmonary hazards. Yang et al.^55^ hypothesized a pancreas–lung axis: Exosomes, which contain proinflammatory mediators, are delivered through the axis to the circulation and distant lung regions.

### 11.1 Fluid resuscitation in AP

Fluid resuscitation is widely recommended for the management of AP.^168^, ^174^ However, there are some disputes and contradictions. Patients receiving aggressive intravenous fluid therapy have a greater risk for ARDS; however, there is no significant change in the incidence of other complications, such as systemic inflammatory response syndrome and persistent organ failure.^175^ Additionally, the studies to date have several limitations: patients are at increased risk for ARDS from excessive intravenous fluid, but these patients may also simply be sicker. In an experimental SAP model, fluid resuscitation ameliorated hemodynamic disorders by altering the expression of aquaporins (AQPs) in the distal colon.^176^ AQPs are integral membrane proteins that belong to the major intrinsic protein family. Additionally, after the inhibition of AQP‐9, the activation of NF‐κB signaling is downregulated, and the infiltration of CD68^+^ and CD11b^+^ cells is reduced in inflammatory tissues.^172^ Moreover, rivastigmine relieves oxidative stress, inflammation, and apoptosis via NF‐κB signaling to ameliorate lung injury.^177^

### 11.2 Inflammation and infection in AP

The use of glucocorticoids in AP treatment is controversial. There is no clear definition for the quantitation of doses used in critical illness. A daily hydrocortisone equivalent between 500 and 1500 mg is equivalent to a moderate dose.^178^, ^179^, ^180^ However, glucocorticoids are the most commonly used drugs for the treatment of ARDS caused by SAP and can effectively reduce mortality. Even so, caution is required with the use of glucocorticoids. Glucocorticoids have a suppressive effect on the immune system. When the body has a severe bacterial infection, glucocorticoids should not be used indiscriminately until effective antibiotics are used to control the infection. SAP is usually accompanied by an altered gut microbiota, which is related to the development of ARDS. However, preventive parenteral antibiotherapy is not associated with a reduction in the aforementioned incidence.^181^, ^182^ Additionally, as protective agents for the gastrointestinal mucosa, PPIs are commonly used. However, a meta‐analysis and systematic review revealed that these agents did not significantly affect outcomes, with the exception of ARDS.^183^, ^184^

### 11.3 Prediction model of AP

SAP patients tend to have longer ICU stays, and their clinical data are more complicated, which is particularly stressful for clinical work. Therefore, screening a small number of clinical factors and constructing a relatively simple prediction model will be helpful in clinical practice. Li et al.^185^ built a prediction model using four clinical indicators (heart rate, respiratory rate, serum calcium, and urea nitrogen) to predict ARDS in patients with AP, which was as accurate as the bedside index for severity in AP but simpler. Among the factors, serum calcium was identified as an important predictor, indicating a potential new strategy for the management of AP. Similarly, Lin et al.^186^ developed an easy‐to‐use nomogram to predict SAP‐related ARDS that included a lower PaO_2_:FiO_2_ ratio and platelet count and higher lactate dehydrogenase, creatinine, and procalcitonin levels were as independent risk factors. Patients with pancreatitis and kidney damage are more likely to have lung damage. Through kidney–lung crosstalk, acute kidney injury has a negative effect on the lungs, and the combination of these two complications significantly increases mortality and reduces prognosis.^187^, ^188^ Therefore, a nomogram offers support for timely prevention and intervention strategies through the use of a simplified model consisting of four variables, including intra‐abdominal pressure, shock, C‐reactive protein, and lactate dehydrogenase.^189^ The intra‐abdominal pressure was consistent with that reported in previous studies. According to the National Inpatient Sample database of adult AP patients, AP patients with abdominal compartment syndrome have elevated inpatient mortality and all major complications, including ARDS (16.06% vs. 0.15%).^190^ SAP is a clinically critical disease that rapidly progresses, not only to cause local disorders but also to cause damage to distant organs. On the basis of clinical observations, we can evaluate disease severity not only from the new predictive model but also from chest CT findings at the time of initial diagnosis. Song and Xiao^191^ concluded that measurements of pleural effusion and the number of lobes involved in pulmonary consolidation on chest CT exhibits certain predictive value for the development of diseases.

There are many integration points between artificial intelligence and the medical fields, such as medical imaging, auxiliary diagnosis, disease prediction, and science education in disease research.^192^, ^193^ At present, research has focused on the artificial intelligence recognition for disease incidence prediction and severity judgment. Modern medical image sources, including B‐ultrasound, CT, and magnetic resonance imaging (MRI), provide diagnostic and treatment data, leading to the development of methods compatible with various types of machine learning algorithms. The artificial intelligence system developed by some companies has been applied in some hospitals and is a powerful auxiliary medical diagnostic tool. In view of the severity and deterioration of AP and SAP disease, timely and effective intervention, in which early judgment is the key element, is the focus of disease treatment. Zhang and Pang^194^ used 31 features with significant differences between groups with and without ARDS in the training set for modeling. Four models, including Support Vector Machine, Ensembles of Decision Trees, Bayesian Classifier, and nomogram models, were constructed and optimized. The Ensemble of Decision algorithm achieved the highest accuracy and precision. In addition, Zhang et al.^195^ constructed Random Forest, Support Vector Machine, Decision Tree, eXtreme Gradient Boosting, and Artificial Neural Network to predict the severity of ARDS. The eXtreme Gradient Boosting model exhibited the best effect in the prediction of binary classification, whereas of all the models for severity, the Artificial Neural Network has the best accuracy. Therefore, constructing a relative prediction model has great potential for the treatment of AP.

## 12 SEPSIS

**FIGURE 7:**
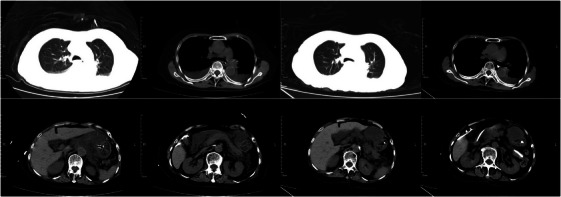
Systemic computed tomography (CT) imaging changes associated with sepsis. Imaging changes before and after treatment in an 81‐year‐old female patient with sepsis (severe acute pancreatitis [SAP] and biliary tract stones).

Sepsis is a serious complication of SAP (Figure 7). Additionally, sepsis and septic shock are common critical illnesses in emergency and ICUs, with high mortality rates and impacts on patients' quality of life. Sepsis tends to develop rapidly, and some cases at the onset of sepsis are hidden. Although there are better diagnostic and treatment techniques and monitoring measures, the incidence and fatality rates of sepsis remain high, which is a prominent problem in the global medical community.^196^, ^197^, ^198^, ^199^ Epidemiological survey data revealed that the annual incidence of severe sepsis is approximately 6‰, and approximately 50% of patients with severe sepsis need to be admitted to ICUs. The mortality rate of such patients is greater than 20%, and the mortality rate of septic shock patients is greater than 50%. According to Sepsis 3.0,^200^ the diagnosis of sepsis must meet the requirements of (1) an uncontrolled host response, (2) the presence of pathogens, and (3) organ dysfunction.

Sepsis is a common risk factor for ARDS. Five variables, namely, pneumonia, local infection, septic shock, the sequential organ failure assessment (SOFA) score, and the nonpulmonary SOFA score, were found to be independent risk factors for ARDS in sepsis patients. In addition, pneumonia is associated with increased severity of ARDS.^201^ Patients with sepsis‐induced ARDS are reported to have a poorer prognosis than those without ARDS.^202^ Therefore, an effective prediction model would be helpful in clinical practice. Xu et al.^203^ developed a prediction model with 13 clinical indices to predict ARDS risk in patients with sepsis, which had favorable discriminatory ability for prediction and was better than the sequential organ failure assessment score and simplified Acute Physiology Score II scoring systems. Moreover, six factors with a consistent and statistically significant association with ARDS, including the sequential organ failure assessment score, Acute Physiology and Chronic Health Evaluation II score, incidence of pulmonary sepsis, smoking status, pancreatitis status, and C‐reactive protein level, were identified.^204^

There have also been breakthroughs in some innovative treatments for sepsis. In induced (cecal ligation and puncture) acute lung injury, melatonin treatment alleviated inflammatory damage, and this effect was strongly correlated with optineurin‐related mitophagy.^205^ In addition, Shimizu et al.^206^ found that extracellular cold‐inducible RNA‐binding protein can downregulate GPX4 expression and increased lipid ROS levels, which may be conducive to the ferroptosis in sepsis‐induced acute lung injury. This research presents a novel therapeutic approach for ferroptosis in sepsis. Additionally, some breakthroughs in research on ferroptosis have been made in traditional Chinese medicine. Ginseng is a valuable Chinese herbal medicine that has a wide range of medical functions. First, as a primary component of ginseng, Panaxydol can reduce pulmonary edema and inflammation, and inhibit ferroptosis in mouse models of lipopolysaccharide (LPS)‐induced acute lung injury. In addition, Panaxydol may suppress ferroptosis in the human bronchial epithelial cells through the Keap1‐Nrf2/HO‐1 signaling pathway.^207^, ^208^, ^209^

## 13 CONCLUSION AND PROSPECTS

ARDS is a clinical syndrome of acute hypoxic respiratory failure caused by intrapulmonary factors or extrapulmonary factors. During the development of ARDS, the main alteration is the increased permeability of alveolar capillaries to fluid, protein, neutrophils, and other blood cells. The different etiologies and pathologies of ARDS reflect different clinical manifestations, and clinicians need to adjust the treatment plan by combining various clinical manifestations. In this review, according to different etiologies, we discuss the latest research progress and treatment strategies for ARDS.

The Berlin definition of ARDS guides ventilator management and therapeutic decisions, especially for severe ARDS, and VV‐ECMO has become an essential treatment, particularly when conventional MV fails to provide adequate treatment.^210^, ^211^, ^212^ During the spread of pneumonia, there was sufficient justification to use ECMO in patients with very severe COVID‐19‐related ARDS, and the mortality rate decreased to approximately 35%.^213^, ^214^, ^215^ However, in contrast to these studies, Karagiannidis et al.^216^ found that, of all 3397 enrolled COVID‐19 patients supported with VV‐ECMO in Germany, in‐hospital mortality was as high as 68%. Differences in mortality across different studies are difficult to interpret. However, VV‐ECMO presents challenges, including coagulation disorders, infections, and organ dysfunction. Thus, careful patient selection, and a strict management system are essential to optimize outcomes and minimize adverse effects of VV‐ECMO.^216^, ^217^, ^218^, ^219^, ^220^

In addition to the above MV treatments, depending on the different etiology, systemic support therapy with fluid resuscitation, glucocorticoids, and antibiotics is also essential. During the follow‐up, anxiety, depression, diffusion impairment, and dysmobility have been reported as causes of concern.^221^, ^222^ Critical care remains an active area of research. It is critical that follow‐up treatment after discharge should pay attention to many forces.

## AUTHOR CONTRIBUTIONS

Rongli Xie, Dan Tan, and Boke Liu drafted the original manuscript. Guohui Xiao and Fangchen Gong provided the clinical data. Qiyao Zhang, Lei Qi, Sisi Zheng, and Yuanyang Yuan revised the original manuscript. Zhitao Yang, Ying Chen, Jian Fei, and Dan Xu reviewed the literature, and conceived and supervised the study. All of the authors read and approved the final manuscript.

## CONFLICT OF INTEREST STATEMENT

The authors declare no conflicts of interest.

## ETHICS STATEMENT

Not applicable.

## Data Availability

The datasets analyzed during the current study are available from the corresponding author upon reasonable request.
